# *Tcap* Deficiency in Zebrafish Leads to ROS Production and Mitophagy, and Idebenone Improves its Phenotypes

**DOI:** 10.3389/fcell.2022.836464

**Published:** 2022-03-15

**Authors:** Xiaoqing Lv, Rui Zhang, Ling Xu, Guangyu Wang, Chuanzhu Yan, Pengfei Lin

**Affiliations:** ^1^ Department of Neurology and Research Institute of Neuromuscular and Neurodegenerative Diseases, Qilu Hospital, Cheeloo College of Medicine, Shandong University, Jinan, China; ^2^ Department of Neurology and Research Institute of Neuromuscular and Neurodegenerative Diseases, Qilu Hospital, Shandong University, Jinan, China

**Keywords:** LGMD2G, TCAP, mitophagy, idebenone, zebrafish, ROS

## Abstract

Limb-girdle muscular dystrophy 2G (LGMD2G) is a subtype of limb-girdle muscular dystrophy. However, the disease’s mechanisms are still not fully understood, and no established therapeutic targets have been found. Using a morpholino-based knockdown approach, we established an LGMD2G zebrafish model. In this study, we found that the ROS level increased in LGMD2G zebrafish. The expression of the mitophagy-related protein BNIP3L, LC3A-II/LC3A-I, and LAMP1 were increased in LGMD2G zebrafish. The oxygen consumption rate and citrate synthase expression was significantly decreased. Thus, mitophagy was presumed to be involved in the LGMD2G to reduce ROS levels. Then, we administered vitamin C, coenzyme Q10, idebenone, metformin, or dexamethasone to rescue LGMD2G in zebrafish. Idebenone reduced the curly tail phenotype and ROS level. Also, it reduced BNIP3L expression in LGMD2G zebrafish models and improved their motor function. In conclusion, mitophagy might be involved in the LGMD2G, and idebenone ameliorated LGMD2G by downregulating ROS level.

## Introduction

Limb-girdle muscular dystrophy 2G (LGMD2G) is a subtype of limb-girdle muscular dystrophy caused by nonsense or frameshift mutations in *TCAP* (Lv et al., 2020). *TCAP* encodes telethonin, a 19 kD protein located in the periphery of Z-discs (Zhang et al., 2009).

Studies examining the pathogenesis of LGMD2G are rare. In 2009, Zhang and his colleagues reported disrupted formation of the T-tubule system in LGMD2G zebrafish models (Zhang et al., 2009). Their study also indicated that stretch force induces variable expression, and *tcap* expression is negatively regulated by integrin-link kinase. In 2010, a *tcap* knockout mouse model was generated and showed a dystrophic phenotype comparable to that of patients with LGMD2G (Markert et al., 2010). In 2013, Ibrahim et al. reported another mouse model. They investigated this gene function in cardiomyocytes, suggesting that telethonin is a critical, load sensitive regulator of t-tubule structure and function (Ibrahim et al., 2013). However, the disease’s mechanisms are still not fully understood, and no established therapeutic targets have been found.

In our previous study in 2020, we observed a disturbing focal sarcomere architecture and abnormal autophagolysosomes, presumably of mitochondrial origin (Lv et al., 2020). Another study revealed intermyofibrillary mitochondrial accumulation and the accumulation of abnormal mitochondria with paracrystalline inclusions in patients with LGMD2G (Paim et al., 2013). These facts hit us those mitochondria dysfunction might exist in LGMD2G. Mitochondrial abnormalities have been observed in individuals with LGMD2B (Liu et al., 2016). Mitophagy has been discovered in the *mdx* mouse model (Kuno et al., 2018), and it might be involved in the pathology of muscular dystrophies (Sebori et al., 2018). In another study, reactive oxygen species (ROS) levels were increased in the muscle of mdx mice models. It illustrated that increased expression of autophagy/mitophagy-related genes and autophagic flux reduces ROS levels in the muscle of mdx mice and thus improved the muscular pathology and physical strength of the mdx mice (Sebori et al., 2018). However, the ROS level in LGMD2G patients and zebrafish has not been studied yet.

In this study, we established an LGMD2G zebrafish model to study the levels of ROS, mitochondrial autophagy-related proteins, citrate synthase (a specific mitochondria marker), and LAMP a lysosome marker (a lysosome marker) in zebrafish with LGMD2G. We found increased expression of the mitophagy-related protein BNIP3L, lysosome marker LAMP1, and LC3A-II/LC3A-I in this zebrafish model. We also found decreased expression of citrate synthase in LGMD2G. Additionally, the oxygen consumption rate (OCR) of the LGMD2G zebrafish decreased, which might be attributed to the reduced number of mitochondria. The mitochondrial DNA (mtDNA) copy number was increased in patients with LGMD2G and zebrafish models. Idebenone reduced the ROS production. Furthermore, it restored the expression of BNIP3L and citrate synthase, mtDNA copy number, and improved their motor function and curly tail phenotype of LGMD2G zebrafish.

## Methods

### Zebrafish Husbandry and Embryo Culture

Adult wild-type zebrafish (AB strain, China zebrafish resource center, China) were housed on a 14 h light/10 h dark cycle with pH 7.5–8.0 and a water conductivity of 980–1,000 μS/cm at 28°C. The animals were fed twice with *Artemia*. We placed breeding pairs in a partitioned breeding tank (Tecniplast) to prevent egg predation at night. The following day, the partition was removed, and the onset of light-triggered spawning. Zebrafish embryos and larvae were cultured in a nonCO_2_ incubator on a 14 h light/10 h dark cycle at 28°C. We injected the *tcap* morpholino (MO) into zebrafish eggs at the one-four cell stage. Four hours after the MO injection, we removed unfertilized eggs. Then, we used E3 medium to wash fertilized eggs several times. Fifty of these fertilized eggs were then incubated in each dish with E3 solution containing 0.1, 0.05, and 0.025 μM or no idebenone for 36 h.

### Morpholino Oligonucleotides

The morpholino oligonucleotides were produced by Gene Tools, LLC (Corvallis, OR, United States). The methods for morpholino preparation and injection were conducted as previously described (Nasevicius and Ekker, 2000). The sequence of *tcap* morpholino (MO) complementary to the translation-blocking target was 5′-CAG​GAC​TGA​GCA​AAC​CTG​CAT​CTT​C. We designed a 5-mispair oligo for the morpholino designed above as a specificity control morpholino (COMO): 5′- CAc​GAa​TGA​GaA​AAC​CTc​CAT​aTT​C.

### Immunostaining of Zebrafish

The immunostaining methods were described in our previous article (Hightower et al., 2020). We used the F59 antibody (Developmental Studies Hybridoma Bank, DSHB) at a 1:50 dilution. Alexa Fluor-conjugated secondary antibodies were used (Invitrogen). After staining, Zebrafish were imaged using a microscope (Olympus BX51, Japan).

### Metabolic Measurements

The OCR was measured using an XF24 Extracellular Flux Analyzer (Seahorse Biosciences). Three zebrafish larvae at 5 h postfertilization (hpf) (*n* = 3–4 per group) or one zebrafish larva at 48 hpf were placed in each well of a 24 well islet microplate, and an islet plate capture screen was placed over the measurement area to ensure that the larvae remained in the center of the well. Measurements were recorded to establish the basal OCR and ATP-linked respiration (Stackley et al., 2011).

### Behavioral Analysis

At 5 days postfertilization (dpf), six embryos from each group were used to evaluate locomotor behavior. Zebrafish were placed into individual wells of a 6-well plate, and swimming behavior was recorded by DanioVision software for 15 min in the dark. After tracking, the software showed the movement locus of zebrafish and recorded the speed of swimming and distance. Heatmaps, swimming distance, and velocity data were generated using DanioVision software.

### qPCR

The mtDNA copy number was analyzed using qPCR. For zebrafish, the forward primer 5′-CCA​CTT​AAT​TAA​CCC​CCT​AGC​C -3′ and reverse primer 5′-ATG​TTT​GTG​GGG​GTA​GAC​CA -3′ for *ND1* encoded in mtDNA were used to amplify the mtDNA. The forward primer 5′-CGC​CTG​AAA​ACT​ACG​TTC​TAC​AC -3′ and reverse primer 5′-ACT​TTC​GGA​GTG​GCT​GAA​AA -3′ for *B2M* were used to amplify the nuclear DNA product. For humans, the forward primer 5′- CAG​CCC​ATG​ACC​CCT​AAC​AG -3′ and reverse primer 5′- TAC​ATC​GCG​CCA​TCA​TTG​GT -3′ for *COX3* encoded in mtDNA were used to amplify the mtDNA. The forward primer 5′- ATG​GTG​AGC​TGC​GAG​AAT​AGC -3′ and reverse primer 5′- GGC​TTC​CTT​TGT​CCC​CAA​TCT​G -3′ for *Actb* were used to amplify the nuclear DNA product. Relative differences in copy number were quantified by analyzing both the difference in threshold amplification between mtDNA and nuclear DNA [delta C(t) method] and a standard curve of a reference template.

### Reactive Oxygen Species Measurement

To evaluate distribution of ROS content, embryos were washed with embryo water twice after exposure, then 2′, 7′-Dihydrodichlorofluorescein diacetate (H2DCFDA) was added at a final concentration of 30 µM, and incubated for 1 h at 28°C in the dark. After staining, Zebrafish were imaged using a microscope (Olympus BX51, Japan).

### Western Blotting Analysis

Proteins were extracted from muscle samples of patients or twenty 48 hpf zebrafish larvae. Western blot analysis was performed as previously described (Lv et al., 2020). The membranes were reacted with antibodies against microtubule-associated protein 1 light chain 3 alpha (LC3A) (18722-1-AP; WUHAN SANYING, Wuhan, China) at a 1:1,000 dilution, BNIP3L (12986-1-AP; WUHAN SANYING) at a 1:1,000 dilution, citrate synthase (ab96600, Abcam, Cambridge, United Kingdom) at a 1:1,000 dilution, and LAMP1 (ab24170, Abcam, Cambridge, United Kingdom) at a 1:1,000 dilution. Detection was accomplished using secondary antibodies. Statistical analyses of LGMD2G zebrafish models were performed using Student’s unpaired t test with GraphPad Prism 8.2.1 software. Unless indicated otherwise, a *p* value < 0.05 was considered statistically significant.

### Muscle Pathology and Ultrastructural Examination

Muscle pathology of patients was analyzed as previously described (Lv et al., 2020). Routine histological and histochemical procedures, including hematoxylin and eosin (HE) staining, modified Gomori trichrome (MGT) staining, nicotinamide adenine dinucleotidetetrazolium reductase (NADH-TR) staining, cytochrome c oxidase (COX) staining, succinate dehydrogenase (SDH) staining, and SDH-COX double staining following incubation at a pH of 4.3 and 10.4, and acid phosphatase (ACP) staining, were performed. The primary antibody used for immunohistochemistry was P62 (Abcam) at a 1:100 dilution. A control muscle specimen collected from healthy people was labeled for comparison between the patient and control sections for each sample.

Patient and zebrafish biopsied muscle specimens were used for the ultrastructural examination described in a previous study (Lv et al., 2020).

## Results

### Muscle Pathology and Ultrastructural Observation

**FIGURE 1:**
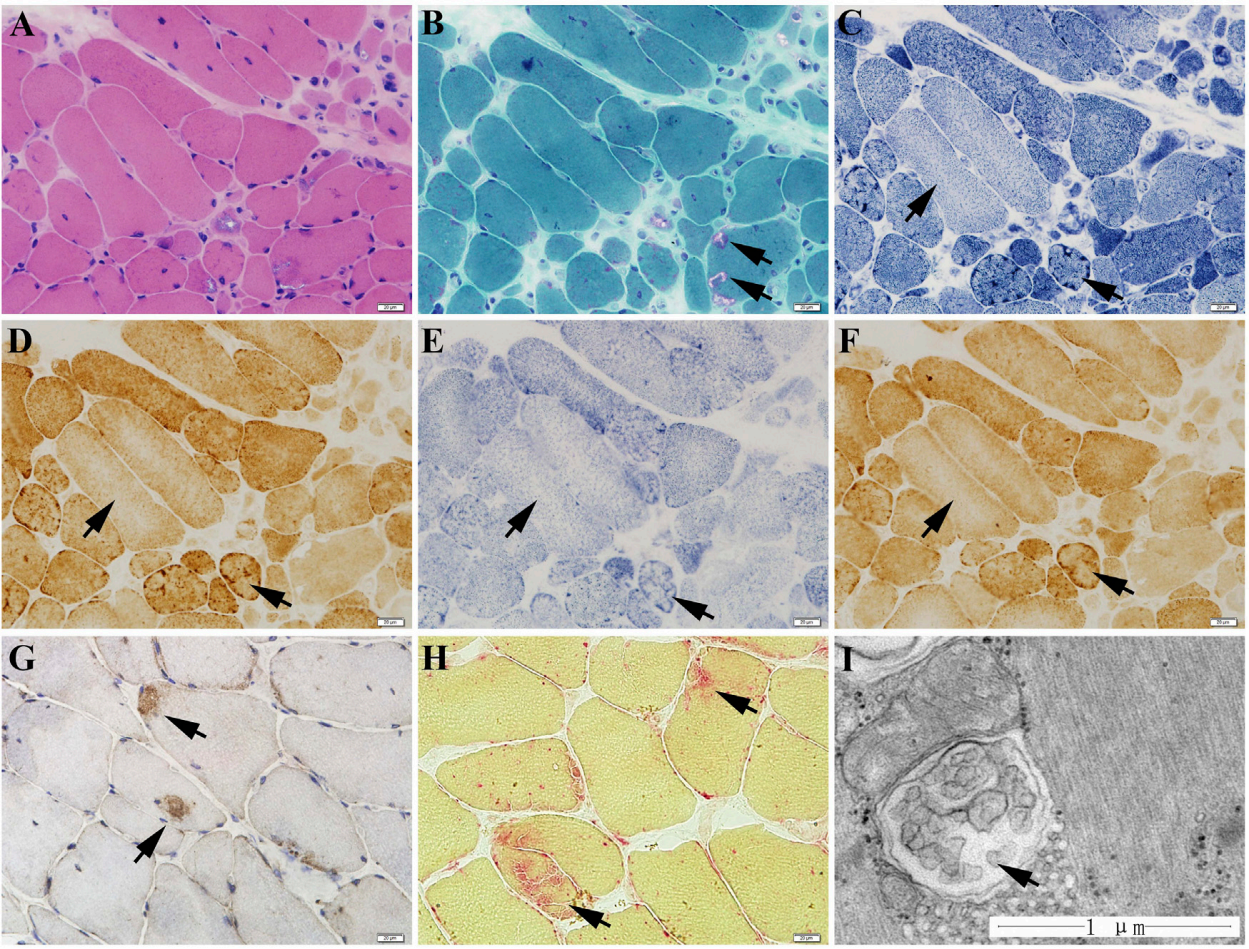
The muscle biopsy specimen from LGMD2G patient. **(A)** HE staining showed obvious variation in fiber size. **(B)** MGT staining showed scattered rimmed vacuoles. No ragged red fiber was found in MGT staining. **(C–E)** The presence of high enzyme activities for NADH **(C)**, COX **(D)**, and SDH **(E)** staining at the periphery of sarcoplasm and the decreased enzyme activities in the center of sarcoplasm. **(F)** No blue fibers in SDH-COX double staining. **(G)** P62 staining showed scattered P62-positive muscle fibers. **(H)** Increased acid phosphatase enzymatic activity (black arrows). **(I)** Ultrastructural examination and muscle pathology of patients with LGMD2G.

The muscle biopsy specimen from LGMD2G patient showed obvious variation in fiber size (Figure 1A), scattered rimmed vacuoles (Figure 1B). The most interesting finding is that we observe abnormal mitochondria distribution, which is illustrated by the presence of high enzyme activities for NADH, SDH, and COX staining at the periphery of sarcoplasm and the decreased enzyme activities in the center of sarcoplasm (Figures 1C–E). No blue fibers were found in SDH-COX double staining (Figure 1F). We also observed scattered P62-positive muscle fibers (Figure 1G) and increased acid phosphatase enzymatic activity (Figure 1H).

The ultrastructure observation of the LGMD2G patient showed an abnormal autophagosome, presumably attributed to mitophagy because of its double membranes (Figure 1I).

### Generation of Tcap Knockdown Zebrafish

**FIGURE 2:**
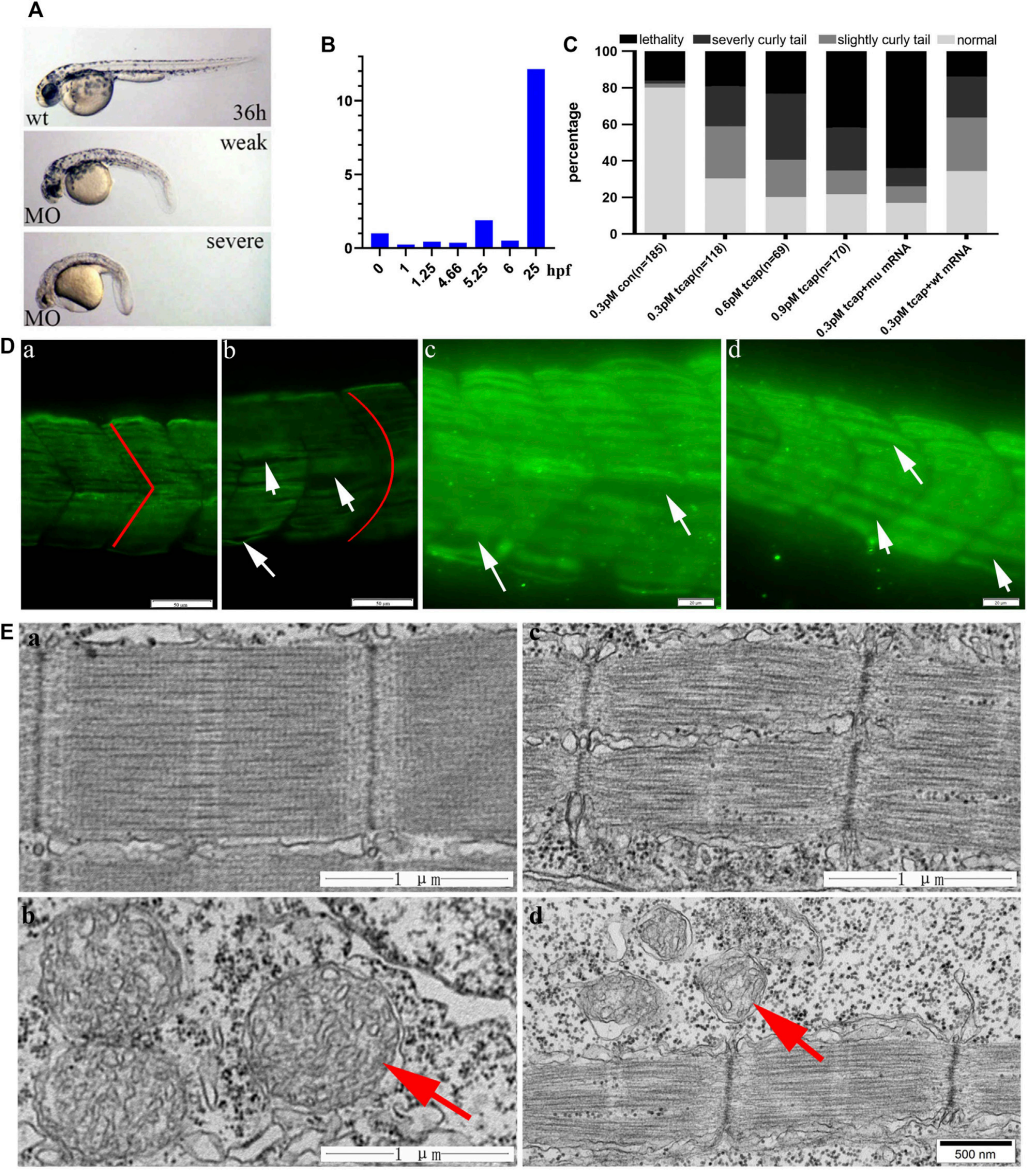
**(A)** MO-tcap results in specific phenotypes: images of 36 hpf wild-type zebrafish and weak and severe phenotypes of the LGMD2G zebrafish model. **(B)** Tcap is expressed at an early stage of embryonic development. **(C)** Dose-dependent effect of MO-Tcap. Coinjection of the wild-type human TCAP mRNA reduced the percentage of the animals with the phenotype at 0.3 pmol, while the mutant TCAP mRNA did not. **(D)** Tcap deficiency causes muscular dystrophy-like phenotypes. The anti-F59 antibody stained muscle fibers in green. COMO fish presented a V-shaped myoseptum and normal sarcomere architecture (red line in a). Tcap knockdown zebrafish exhibited a U-shaped myoseptum (red line in b) that was discontinuous or missing in some regions (white arrows in c and d), and the myofibers grew through adjacent somites. Scale bar, 20 mm. **(E)** Electron microscopy images of control zebrafish (a and b) and tcap knockdown zebrafish (c and d). Red arrows indicate normal mitochondria in b and dysmorphic mitochondria in d.

We knocked down telethonin expression using MO-*tcap* to generate LGMD2G zebrafish model. According to the criteria reported by Zhang et al. (2009), four types of LGMD2G zebrafish models exist: normal, weak (slightly curled tail), severe (severe curled tail) and lethal at 36 hpf (Figure 2A). Zebrafish *tcap* is expressed from the 21-somite stage (Zhang et al., 2009). This article shows that *tcap* is expressed at an early stage of embryonic development (Figure 2B). Injection of 0.3 pmol MO-*tcap* resulted in 33 embryos (*n* = 118) presenting weak phenotypes and 25 presenting severe phenotypes (Figure 2C). Injection of 0.6 pmol MO-*tcap* resulted in 14 embryos (*n* = 69) presenting weak phenotypes and 25 presenting severe phenotypes. Injection of 0.9 pmol MO-*tcap* resulted in 22 embryos (*n* = 170) presenting weak phenotypes and 40 presenting severe phenotypes. A U-shaped myoseptum was observed in LGMD2G zebrafish, but a V-shaped myoseptum was observed in the control zebrafish (Figure 2D), consistent with a previous study (Zhang et al., 2009). In some areas, myofibrils from neighboring somite segments were separated (white arrows in Figure 2D).

After coinjection of 30 pg of wild-type human *TCAP* mRNA with 0.3 pmol MO-*tcap* at the one-four cell stage, the percentage of larvae exhibiting severe phenotypes was reduced from 36.23 to 22.41%. However, coinjection of 30 pg of the mutant human *TCAP* mRNA with 3 pmol of MO-*tcap* at the one-four cell stage increased the percentage of lethal embryos from 23.19 to 64.00% (Figure 2C).

The ultrastructural examination of control zebrafish is shown in Figure 2E, a, and b. *Tcap* knockdown zebrafish exhibited destroyed M-lines (Figure 2E, c) and abnormal mitochondria (Figure 2E, d).

### Oxygen Consumption Rate of Zebrafish

**FIGURE 3:**
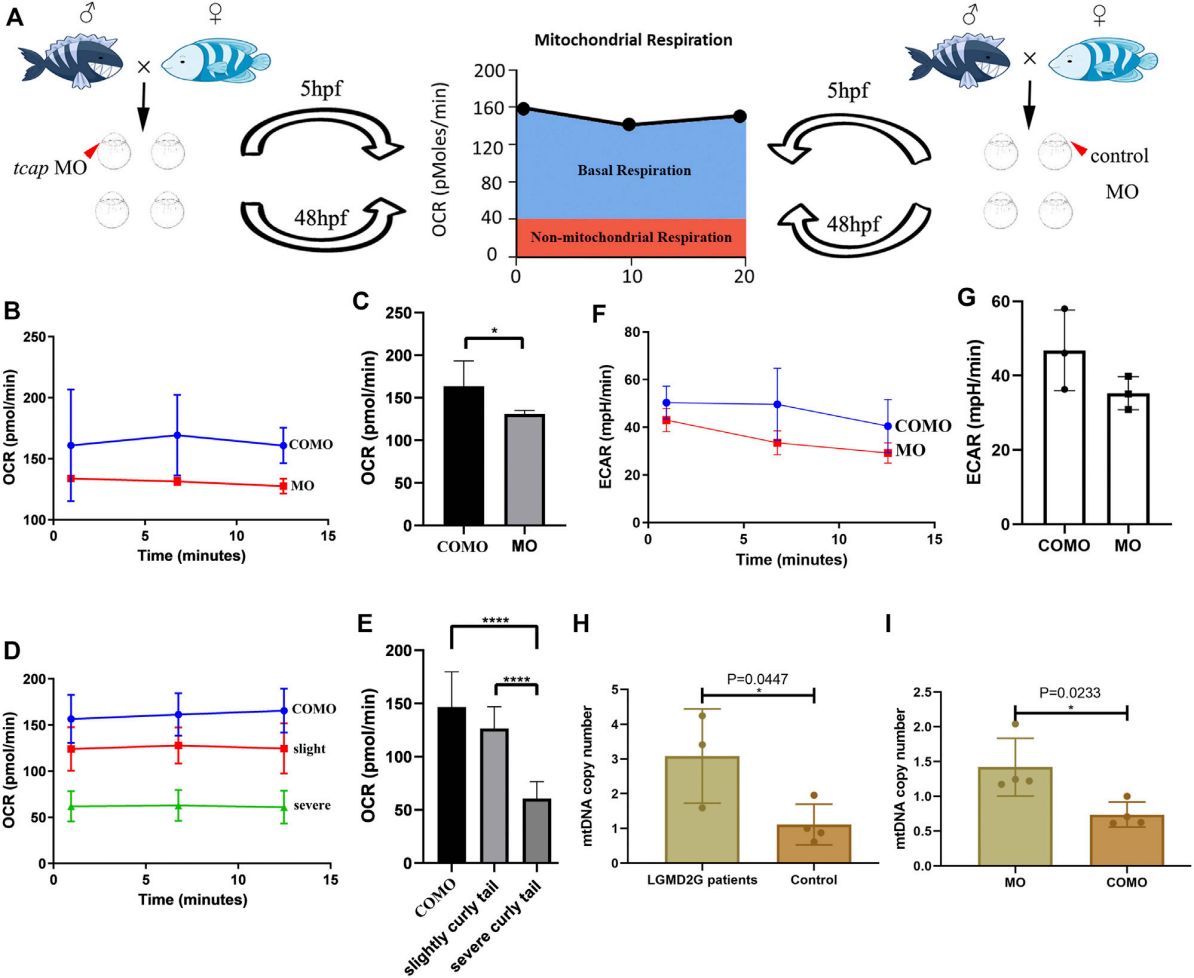
**(A)** Cartoon representation of how the Seahorse bioanalyzer displays mitochondrial bioenergetics. **(B,C)** OCR of control subjects (three zebrafish larvae in each group, four groups in total) and *tcap* knockdown zebrafish (four groups) at 5 hpf. The OCR of control subjects was significantly higher than that of *tcap* knockdown models of LGMD2G. An unpaired t test was used to compare the MO and COMO groups. * Indicates *p* < 0.05. **(D,E)** Comparison of OCR among control subjects (*n* = 4), *tcap* knockdown zebrafish with the slight phenotype (*n* = 4), and *tcap* knockdown zebrafish with the severe phenotype (*n* = 4). The OCR of control subjects was significantly higher than that of slightly curly tailed or severe curly tailed MO-*tcap* zebrafish. One-way ANOVA (Dunnett’s post hoc test) was used to compare the COMO, slightly curly tail, and severe curly tail groups; ****indicates *p* < 0.001. **(F,G)** ECR of control subjects (three zebrafish larvae in each group, four groups in total) and tcap knockdown zebrafish (four groups) at 5 hpf. The ECAR of control subjects was not significantly higher than that of *tcap* knockdown models of LGMD2G. An unpaired t test was used to compare the MO and COMO groups. **(H,I)** MtDNA copy number in patients with LGMD2G and control subjects **(H)** and *tcap* knockdown zebrafish and control zebrafish **(I)**. Unpaired t test was used for comparisons. * Indicates *p* < 0.05.

We divided zebrafish into two groups: Group 1 included zebrafish injected with COMO; Group 2 included zebrafish injected with MO-*tcap*. At 48 hpf, three phenotypes were observed in Group 2: normal, weak, and severe. We evaluated the OCR in living zebrafish eggs and larvae by moving 5 hpf and 48 hpf COMO zebrafish and MO-*tcap* zebrafish into islet capture plates (Figure 3A). A significant decrease in the oxygen consumption rate was observed in MO-*tcap* zebrafish (Figures 3B–E). No significant difference in ECAR was observed between COMO zebrafish and MO-*tcap* zebrafish; however, the ECAR in COMO zebrafish tended to be higher than that in MO-*tcap* zebrafish (Figures 3F, G).

### Mitochondrial Copy Number Analysis

In humans, the mtDNA copy number was detected in muscle tissue from patients with LGMD2G (*n* = 3) and control subjects (*n* = 4). A significant increase in the mtDNA copy number was observed in patients with LGMD2G (Figure 3H). In zebrafish, the mtDNA copy number was detected in muscle tissue from MO-*tcap* (*n* = 4) and COMO zebrafish (*n* = 4). We also observed a significant increase in the mtDNA copy number in MO-*tcap* zebrafish (Figure 3I).

### Protein Analysis

**FIGURE 4:**
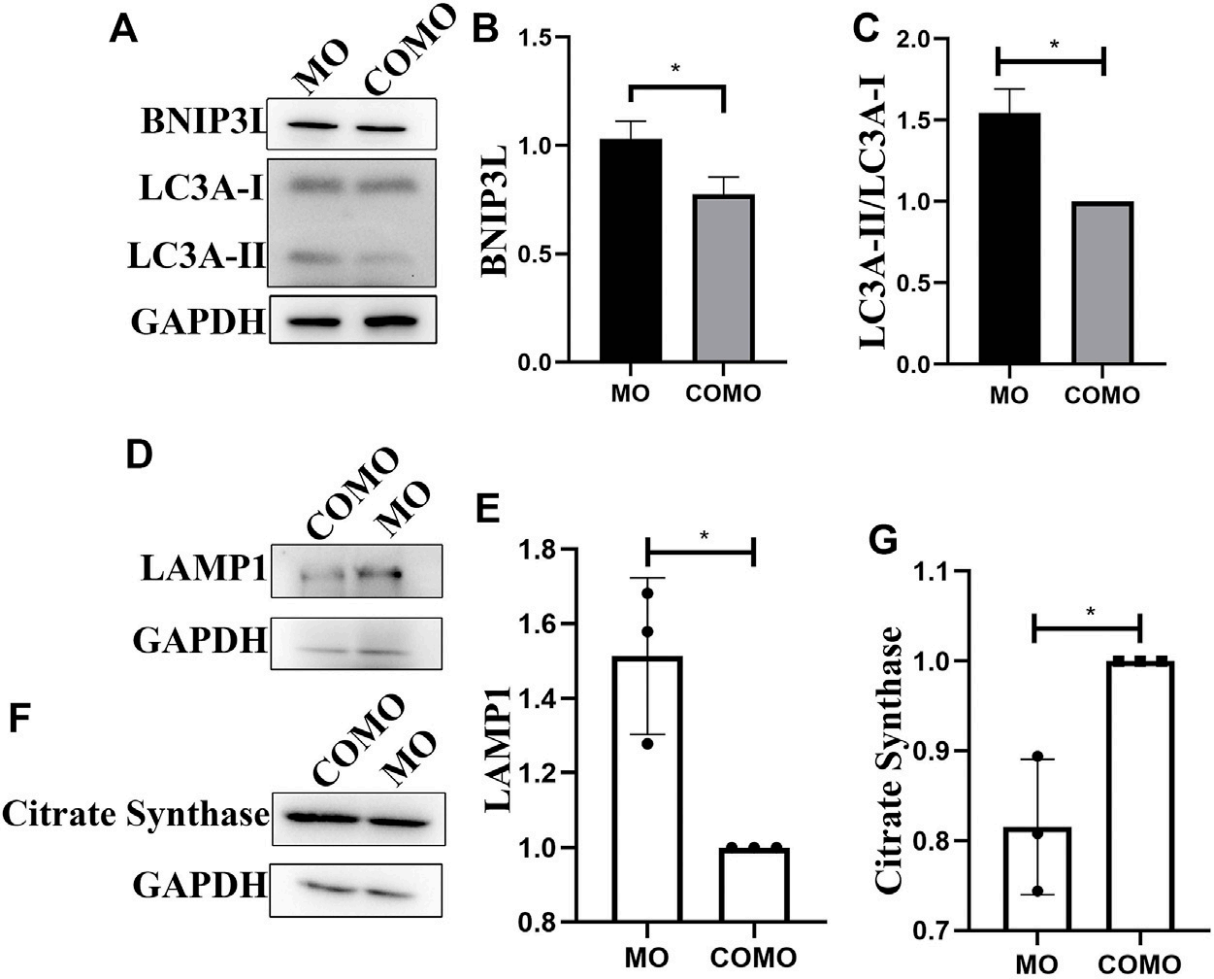
**(A,D,F)** BNIP3L, LC3A, LAMP1 and citrate synthase expression in 48hpf zebrafish. **(B,C,E,G)** Quantification of western blot results from LGMD2G zebrafish models. Values are presented as the means ± SD of 3 separate experiments. Unpaired t test was used for comparisons. *Indicates *p* < 0.05.

BNIP3L is a mitophagy marker, and it interacted with LC3A but not with LC3B (Hanna et al., 2012). As shown in Figure 4, we investigated mitophagy in zebrafish models by performing Western blotting to evaluate the expression levels of BNIP3L, LAMP1, citrate synthase, and LC3A-II/LC3A-I. In the LGMD2G zebrafish model, BNIP3L expression, LC3A-II/LC3A-I, LAMP1 expression was significantly increased compared with the control group (Figures 4A–E). What’s more, we found citrate synthase expression decreased significantly in LGMD2G zebrafish model (Figures 4F, G).

### Idebenone Reduces the Curly Tail Phenotype of LGMD2G Zebrafish Larvae

We administered different concentrations of vitamin C, coenzyme Q10, idebenone, metformin, or dexamethasone to LGMD2G zebrafish larvae. The drug we select those found to be useful in DMD, including metformin, dexamethasone, coenzyme Q10 and idebenone (Werneck et al., 2019), and vitamin C. However, these drugs did not ameliorate the phenotype of LGMD2G zebrafish, except for idebenone.

**FIGURE 5:**
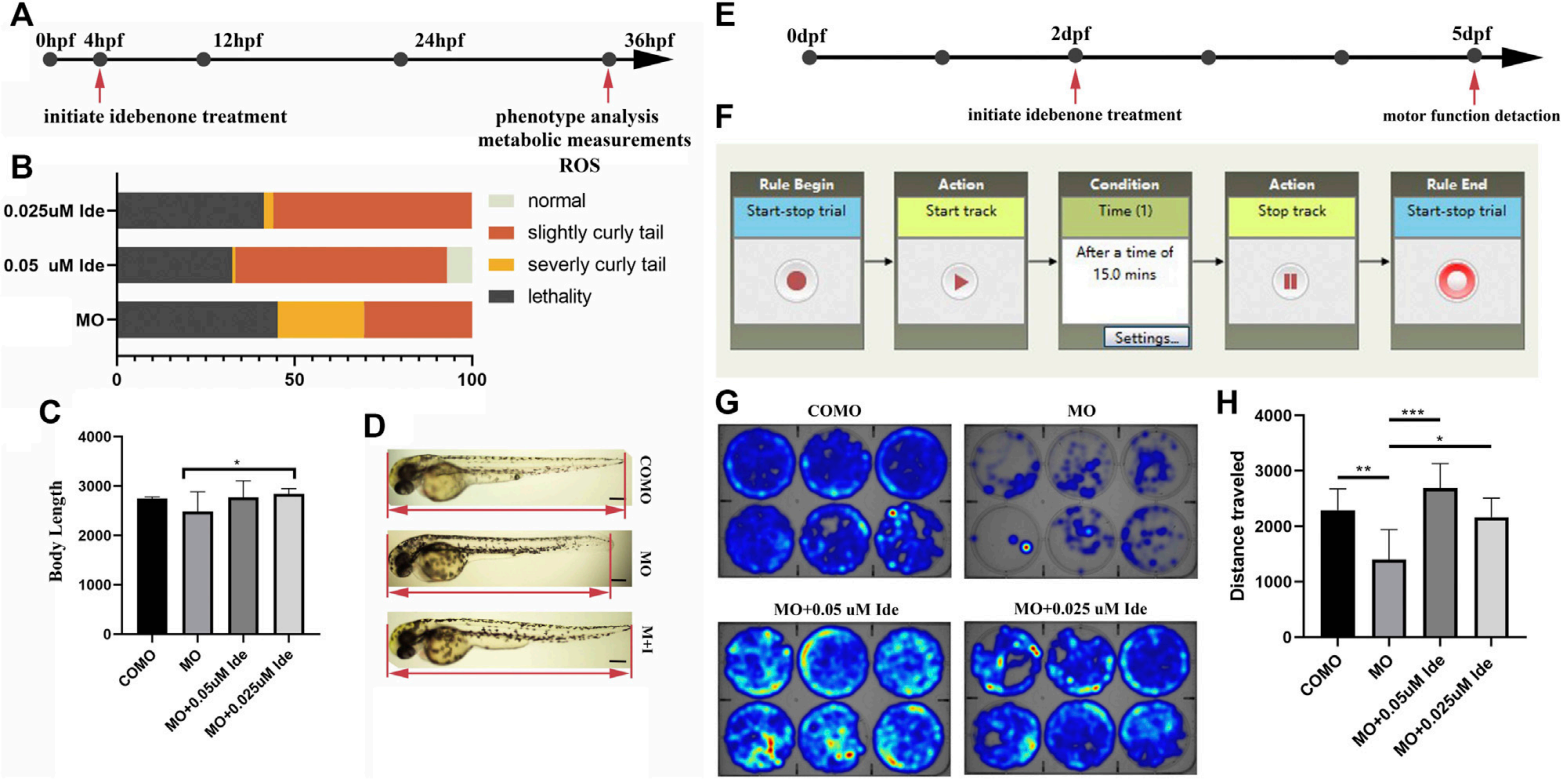
**(A)** Design of the short-term phenotype assay in zebrafish. MO-*tcap* was injected into one-four cell stage embryos. Drug treatments were applied from 4 hpf. At 36 hpf, the phenotype was analyzed. **(B)** Graphic summary of the percentage of normal, slightly curly tail, severely curly tail, and lethality in different classes of LGMD2G zebrafish with or without idebenone treatment. **(C)** Body length analysis of different groups. One-way ANOVA (Dunnett’s post hoc test) was used to compare the MO, MO + 0.05 μM Ide, MO+0.025 μM groups. **(D)** Idebenone restored the body length of LGMD2G zebrafish. M + I, MO + idebenone. **(E)** Design of the short-term swimming activity assay in zebrafish: pairs of wild-type zebrafish were mated, and COMO and MO were injected into 1-4 cell stage embryos. Drug treatments were applied to the progeny in six-well plates (1 embryo per well) beginning at 2 dpf. At 5 dpf, a swimming activity assay was performed. **(F)** The locomotor activity settings for one cycle (15 min) of tracking are shown. **(G)** Representative heatmaps of swimming activity produced by DanioVision comparing COMO-injected, MO-injected, and MO-injected drug-treated fish over 15 min. Heatmap showing the tracking path of six zebrafish from each experimental group. The blue marks indicate the area the zebrafish passed through, and the depth of color represents the cumulative frequency of the tracking path. **(H)** Distance traveled by zebrafish from four different groups (COMO, MO, MO + 0.05 μM Ide, and MO + 0.025 μM Ide). One-way ANOVA (Dunnett’s post hoc test) was used for comparisons among the MO, MO+0.05 μM Ide, and MO+0.025 μM groups. An unpaired t test was used to compare the MO and COMO groups. *p* < 0.05 (*), 0.001 < *p* < 0.05 (**), *p* < 0.001 (***).

After injection of 0.3 pmol of MO-*tcap*, 50 of these fertilized eggs were then incubated in each dish with E3 solution containing 0.05 μM, 0.025 μM or no idebenone up to 36 hpf (Figure 5A). The phenotypes were divided into four types: normal, weak, severe, and lethal at 36 hpf. The administration of 0.05 μM idebenone showed greater efficacy than 0.025 μM (Figure 5B). Then, we analyzed body length (Figure 5C). The body length of LGMD2G zebrafish was 2482.32 μm, shorter than that of the COMO group, namely, 2749.45 μm. Idebenone (0.05 and 0.025 μM) restored the body length of LGMD2G zebrafish larvae to 2767.13 and 2846.17 μm, respectively (Figure 5D).

### Idebenone Improves the Motor Function of the LGMD2G Zebrafish Model

Given the substantial correction of the curly tail phenotype, we hypothesized that the motor function of the LGMD2G zebrafish model might be improved after incubation with idebenone. Using DanioVision software, we developed a dark locomotion assay in 6-well plates to evaluate the motor function of fish (Figures 5E, F).

MO-*tcap*-injected embryos treated with or without idebenone in the water were individually placed in wells of 6-well plates to analyze the distance traveled by fish and velocity at 5 pdf. The mean total swimming distance of LGMD2G zebrafish (*n* = 6) was 1,401.11 mm, shorter than that of COMO zebrafish (*n* = 6), namely, 2291.83 mm. The mean total swimming distances of 0.05 μM (*n* = 6) and 0.025 μM (*n* = 6) idebenone-treated LGMD2G zebrafish were 2689.43 mm and 2165.68 mm, respectively. Idebenone-treated MO zebrafish exhibited a more active swim activity heatmap than the MO-treated cohort (Figures 5G, H).

### Idebenone Increased OCR and ATP Production of the LGMD2G Zebrafish Model

**FIGURE 6:**
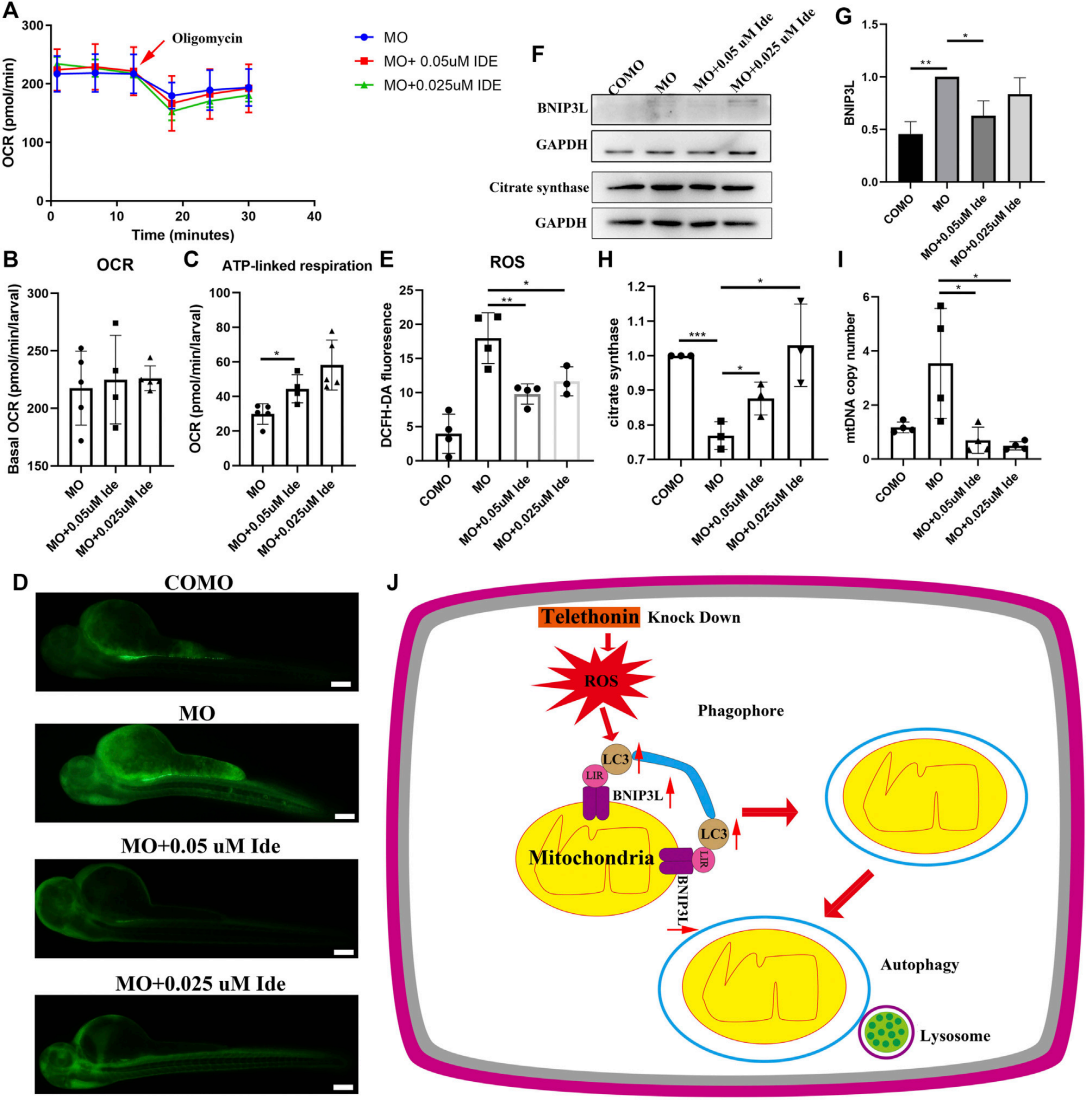
**(A)** OCR of MO subjects (*n* = 5), MO + 0.05uM Ide (*n* = 4), and MO + 0.025uM Ide (*n* = 5) zebrafish at 48 hpf. **(B)** Basal OCR of MO subjects (*n* = 5), MO + 0.05uM Ide (*n* = 4), and MO + 0.025uM Ide (*n* = 5) group. The data represent means ± S.D. **(C)** ATP-linked respiration was studied for all groups. The data represent means ± S.D. *p < 0.05. **(D)** Distribution of fluorescence visualizing ROS in COMO, MO, MO + 0.05uM Ide, and MO + 0.025uM Ide group in 48 hpf. **(E)** Fluorescence intensity of ROS. The data represent means ± S.D. *p < 0.05, **p < 0.01. **(F)** Western blot showing BNIP3L, and citrate synthase levels in control, LGMD2G zebrafish model and drug-treated LGMD2G zebrafish. **(G,H)** Quantification of western blot results from zebrafish. One-way ANOVA (Dunnett’s post hoc test) was used for comparisons with the MO, MO + 0.05 μM Ide, MO + 0.025 μM groups. An unpaired t test was used for comparisons with the MO and COMO groups. *p* < 0.05 (*), 0.001<*p* < 0.05 (**), *p* < 0.001 (***). **(I)** MtDNA copy number in COMO, MO, MO + 0.05 μM Ide, MO + 0.025 μM groups. Unpaired t test was used for comparisons. * Indicates *p* < 0.05. **(J)** Cartoon representation of mitophagy generation after *tcap* knockdown.

We divided zebrafish into three groups: Group 1 included zebrafish injected with MO-*tcap*; Group 2 included zebrafish injected with MO-*tcap* and then treated with 0.05uM idebenone; Group 3 included zebrafish injected with MO-*tcap* and then treated with 0.025uM idebenone. At 48 hpf, the OCR were evaluated in living zebrafish eggs and larvae of these three groups by moving zebrafish into islet capture plates. After idebenone treatment, data illustrated that no significant difference in basal OCR between MO zebrafish, MO + 0.05uM Ide group, and MO + 0.025uM Ide group. However, the basal OCR in idebenone treatment groups tended to be higher than that in MO-*tcap* zebrafish (Figures 6A, B). Oligomycin OCR is used to determine ATP-linked respiration (Bond et al., 2018). A significant increase in the ATP-linked respiration was observed in idebenone treatment zebrafish groups (Figures 6A, C).

### Idebenone Reduces the ROS Production of the LGMD2G Zebrafish Model

ROS levels were demonstrated to increase in the muscle of mdx mice models (Sebori et al., 2018). So we detect ROS level in LGMD2G zebrafish models. ROS staining was performed after exposure to idebenone at 48 hpf, and the fluorescence intensity was calculated using ImageJ (Figures 6D, E). Compared with the control group, ROS content increased significantly in MO-*tcap* group. In addition, the administration of idebenone showed decreased ROS (Figures 6D, E).

### Idebenone Reduces Mitophagy Related Protein Expression, and Restores Citrate Synthase Expression and MtDNA Copy Number

MO-*tcap*-injected zebrafish exhibited an increase in BNIP3L expression. In MO-*tcap*-injected zebrafish treated with 0.05 μM idebenone, BNIP3L expression was decreased to the baseline levels observed in the COMO group (Figures 6F, G). Moreover, idebenone treatment restored citrate synthase expression and reduced mtDNA copy number (Figures 6F, H, I).

In conclusion, these results prove the hypothesis that idebenone reduced the ROS production. Furthermore, it restored the expression of BNIP3L and citrate synthase, mtDNA copy number, and improved their motor function and phenotype of LGMD2G zebrafish.

## Discussion

In this article, we successfully established an LGMD2G zebrafish model by injecting morpholino oligomers. The zebrafish model showed a phenotype mimicking muscular dystrophy characterized by body curvature and reduced swimming ability.

Mitochondrial ultrastructural alterations are observed in individuals with many muscular disorders. For example, Bethlem myopathy caused by *COLA6* mutation is also related to mitochondrial dysfunction and autophagy (Bernardi and Bonaldo, 2013). Inefficient autophagy is related to mitochondrial diseases and collagen VI muscular dystrophies (Zhang et al., 2020). In 2018, ROS level was demonstrated to increase in DMD mouse and increased expression of autophagy/mitophagy-related genes. Autophagic flux reduces ROS levels in the muscle of mdx mice and then improves the muscular pathology and physical strength of the mdx mice (Sebori et al., 2018). It is proposed that damaged or dysfunctional mitochondria are the primary source of ROS in most cells. These types of mitochondria are eliminated by mitophagy. Autophagy insufficiency induced by the knockout of autophagy/mitophagy-related genes causes a significant increase in cellular ROS, suggesting that ROS are liberated from damaged mitochondria that escape mitophagy. Thus, the insufficiency of mitophagy may have a role in the pathology of muscular dystrophies. In the present study, the ROS level of MO-*tcap* zebrafish is higher than control zebrafish. So we then detected the expression of the mitophagy-related protein, BNIP3L. Mitophagy regulates the turnover of damaged and dysfunctional mitochondria (Fivenson et al., 2017). In mammals, mitophagy is mediated by NIP3-like protein X (BNIP3L) during red blood cell differentiation (Youle and Narendra, 2011). BNIP3L interacts with LC3A through a BH3 domain (Novak et al., 2010; Hanna et al., 2012) and is a proapoptotic protein (Chen et al., 1999). BNIP3L transcription is triggered in various cells under hypoxic conditions (Yuan et al., 2017). Nix might primarily regulate basal levels of mitophagy under physiological conditions and induce mitophagy under hypoxic conditions (Yoo and Jung, 2018). In the present study, BNIP3L expression and LC3A-II/LC3A-I were increased significantly in LGMD2G zebrafish models. These facts indicate an impairment of mitochondria. Lysosomes marker LAMP1 also increased significantly in LGMD2G zebrafish model. Muscle pathology of LGMD2G patient showed high acid phosphatase enzymatic activity (Figure 1H, black arrow). The acid phosphatase enzyme is an enzyme of the lysosome. An increase of acid phosphatase enzyme in LGMD2G patients indicates an increase in the lysosome number. These facts indicated mitophagy was involved in LGMD2G zebrafish.

We observed citrate synthases decreased significantly in LGMD2G zebrafish. This fact suggests the mitochondria number is reduced in LGMD2G zebrafish, which might be caused by increased mitophagy. Also, we observed decreased OCR of LGMD2G zebrafish, which a reduced number of mitochondria might cause.

Idebenone, an analog of coenzyme Q10, is an antioxidant. A previous study indicated that long-term treatment with idebenone reduces the annual decrease in FVC%p by approximately 50% in patients with DMD (Servais et al., 2020). Our study showed that idebenone improved the curly tail phenotype, motor dysfunction, and reduced ROS level. What’s more, idebenone restored the BNIP3L expression and citrate synthase expression. Therefore, idebenone reduces ROS in LGMD2G zebrafish and might be a potential therapeutic drug for LGMD2G. According to our data, it is interesting that idebenone-treated MO zebrafish exhibited a more active swim activity and longer body length than COMO zebrafish. A study reported that complex II subunit SDHD is critical for cell growth and metabolism. The respiratory and growth defects caused by SDHD knockout can be partially restored with treatment of idebenone in cells (Bandara et al., 2021). So we suggested idebenone might promote cell growth and development. This theory might explain why idebenone-treated MO zebrafish exhibited a more active swim activity and longer body length than COMO zebrafish.

In this study, we observed an increased mtDNA copy number in patients with LGMD2G and LGMD2G zebrafish models. After treatment with idebenone, mtDNA copy number of LGMD2G zebrafish was restored.

As shown in the present study, after *tcap* knockdown, ROS increased. Dysfunctional mitochondria might produce ROS. Then the expression of LC3A-II/LC3A-I and BNIP3L increased to eliminate dysfunctional mitochondria to reduce ROS. Damaged mitochondria are recognized by phagophores to form autophagosomes. Then, the autophagosome fuses with the lysosome to induce mitophagy (Figure 6J). Our study suggests that idebenone might benefit LGMD2G zebrafish by reducing ROS and supports a new indication for idebenone as a potential LGMD2G therapeutic to be further investigated in a mouse model of LGMD2G.

## Data Availability

The original contributions presented in the study are included in the article/Supplementary Material, further inquiries can be directed to the corresponding author.
